# Effect of docosahexaenoic acid plus insulin on atherosclerotic human endothelial cells

**DOI:** 10.1186/s12950-021-00277-5

**Published:** 2021-02-18

**Authors:** Aysan Eslami Abriz, Reza Rahbarghazi, Alireza Nourazarian, Çıgır Biray Avci, Soltan Ali Mahboob, Maryam Rahnema, Atefeh Araghi, Morteza Heidarzadeh

**Affiliations:** 1 Department of Biochemistry, Higher Education Institute of Rab-Rashid, Tabriz, Iran; 2 Stem Cell Research Center, Tabriz University of Medical Sciences, Tabriz, Iran; 3 Department of Applied Cell Sciences, Faculty of Advanced Medical Sciences, Tabriz University of Medical Sciences, Tabriz, Iran; 4 Department of Biochemistry and Clinical Laboratories, Faculty of Medicine, Tabriz University of Medical Sciences, Golgasht St, Tabriz, 51666-16471 Iran; 5 Department of Medical Biology, Faculty of Medicine, Ege University, Izmir, Turkey; 6 Department of Clinical Sciences, Faculty of Veterinary Medicine, Amol University of Special Modern Technologies, Amol, Iran

**Keywords:** Endothelial cells, Atherosclerosis, Docosahexaenoic acid, Insulin, Palmitic acid, Cell bioactivity

## Abstract

**Background:**

Atherosclerosis is touted as one of the most critical consequences of diabetes mellitus indicated by local inflammation of endothelial cells. The Effect of Omega 3 fatty acids, mainly docosahexaenoic acid (DHA), has been investigated in cells after exposure to high doses of lipids. The current experiment aimed to address the modulatory effects of docosahexaenoic acid and insulin in palmitic-treated human endothelial cells.

**Methods:**

Human umbilical vein endothelial cells were treated with 1 mM palmitic acid, 50 μM insulin, 50 μM docosahexaenoic acid, and their combination for 48 h. Cell survival rate and apoptosis were measured using MTT and flow cytometry assays. The Griess assay detected NO levels. Protein levels of TNF-α, IL-6, and NF-κB were studied using ELISA and immunofluorescence imaging. The expression of genes participating in atherosclerosis was monitored using PCR array analysis.

**Results:**

Oil Red O staining showed the inhibitory effect of DHA and insulin to reduce the intracellular accumulation of palmitic acid. Both DHA and Insulin blunted palmitic acid detrimental effects on HUVECs indicated by an increased survival rate (*p* < 0.05). The percent of apoptotic cells was decreased in palmitic-treated cells received insulin and DHA compared to palmitic-treated group (*p* < 0.05). Based on our data, DHA and Insulin diminished the production of all inflammatory cytokines, TNF-α, IL-6, and NF-κB, in palmitic-treated cells (*p* < 0.05). Similar to these data, NO production was also decreased in all groups treated with insulin and DHA compared to the palmitic-treated cells (*p* < 0.05). PCR array analysis revealed the modulatory effect of DHA and insulin on the expression of atherosclerosis-related genes pre-treated with palmitic acid compared to the control group (*p* < 0.05).

**Conclusion:**

DHA and Insulin could alter the dynamic growth and dysfunctional activity of human endothelial cells after treatment with palmitic acid. Taken together, Omega 3 fatty acids, along with insulin, could dictate specific cell behavior in endothelial cells in vitro.

## Introduction

Atherosclerosis is touted as a leading cause of cardiovascular diseases, leading to a high rate of mortality [1, 2]. Epidemiological studies and clinical examinations showed that obesity and metabolic disorders predispose individuals to atherosclerotic changes. During atherosclerotic changes, the luminal surface of vessels is injured, and atherosclerotic plaques form. The promotion of atherosclerotic changes, in turn, increases subsequent inflammatory response which leads to local thrombosis and ischemic conditions [3]. It is thought that the formation of thrombotic and atherosclerotic thrombi not only increases the risk of the ischemic condition but also promotes the endothelial cells’ injury. Along with these changes, immune cells such as macrophages and T lymphocytes are recruited to the site of atherosclerotic plaques, which in turn exacerbate atherosclerotic injuries [4]. Inside the atherosclerotic plaques, large amounts of low-density lipoprotein existed that promoted further macrophages recruitment and expansion of vessel injuries [5].

The emergence of chronic metabolic disorders, such as type 2 diabetes mellitus (T2DM), was shown to increase the risk of atherosclerotic plaques development [6]. The increase of insulin levels (hyperinsulinemia) intensifies the formation of atherosclerotic plaques in addition to disturbances in lipid metabolism seen under diabetic conditions [7, 8]. Therefore, one could hypothesize that atherosclerotic plaques are developed in response to different stimuli. It is thought that the increased insulin occurs in response to insulin receptors’ sensitivity after the onset of T2DM. In response to these conditions, endothelial cells adopted themselves to decrease the membrane distribution of insulin receptors [9]. Along with these changes, the downstream insulin signaling pathway is ceased, contributing to a decrease of Akt phosphorylation, and inactivation of endothelial nitric oxide (eNOS) coincided with increased palmitoylation rate [10].

In addition to conventional therapies, many attempts have been made to alleviate atherosclerotic plaques by using natural compounds in addition to drugs [11]. The application of omega-3 polyunsaturated fatty acids was shown to decrease the possibility of atherosclerotic changes. For example, all-cis-docosa-4, 7, 10, 13, 16, 19-Hexa-enoic acid (DHA) possess anti-inflammatory properties to decrease the production of cytokines such as TNF-α, IL-1β, and IL-6 while the phosphorylation of eNOS and levels of endothelial NO increases [12]. It was also mentioned that omega-3 compounds such as DHA could decrease the levels of NF-ƙB and endothelial injury. The current experiment was designated to elucidate the therapeutic effects of DHA on atherosclerotic endothelial cells pre-treated with a high dose of insulin. We tried to answer whether DHA could or/not decrease the detrimental effect of palmitic acid under hyper-insulinemia conditions [13].

## Material and methods

### Cell culture protocol

To evaluate the treatment protocol, we selected human umbilical vein endothelial cells (HUVECs). HUVECs were purchased from the National Cell Bank of Iran (NCBI code: C554). The cells were expanded in Dulbecco’s Modified Eagle Medium: Nutrient Mixture F-12 (DMEM/F-12; Gibco) under reasonable condition consisted of 37 °C temperature, 5% CO_2,_ and 95–98% humidity. To induce cell proliferation, culture media is supplemented with 10 10% fetal bovine serum (FBS) and 1% Pen-Strep solution. Cells were detached using 0.25% Trypsin-EDTA solution (Gibco). We enrolled the cells to different analyses between passages 3–6.

### Treatment protocol

HUVECs were allocated into seven different groups as follows; the non-treated Control cells; Pal; DHA, DHA+ Pal; Insulin, Insulin +Pal, and Insulin + DHA + Pal. To induce atherosclerotic changes, cells were treated with a culture medium containing 1 mM Pal (Cat no. P0500-10G, Sigma) and 2% FBS for 48 h. We also incubated HUVECs with 50 μM insulin for the induction of hyperinsulinemia conditions for 48 h. After completion of hyperinsulinemia condition and atherosclerotic changes, we added the 50μMDHA (Cat no. D253425MG, Sigma) to the respective groups and cells allowed to expose to DHA for 48 h.

### Preparation of albumin (Alb)-pal conjugate

To increase the solubility of Pal, we prepared conjugated Alb-Pal composition according to our previously published protocols [14]. In brief, 2.267 ***g*** bovine serum albumin (Sigma-Aldrich) was dissolved in 150 mM NaCl solution and maintained at 37 °C. Then, Pal (30.6 *mg*) was added to the solution and heated up to 70 °C. The mixture was gently stirred at 40 °C for 1 h. The pH was adjusted to 7.4. The final solution was passed through the 0.22-μm pore size micro-filter (Membrane Solution).

### Confirmation of intracellular pal accumulation by oil red O staining

The oil red O staining method was used to evaluate the intracellular deposition of Pal inside the HUVECs. To this end, 3× 10^5^ HUVECs were placed in each well of 24-well plates. After the completion of experiments, the supernatant medium was discarded, and treated cells from different groups were fixed with pre-cooled 4% paraformaldehyde (PFA) solution at 4 °C for 20 min. After that, 200 μl Oil red O solution (0.5% w/v) was added to each well, and plates were kept at room temperature for 20–30 min. Then, cells were washed twice with PBS and imaged by using an inverted microscope. The number of red-colored cells in the group was counted in 10 random serial fields and compared to each other [15].

### MTT assay

200 μl medium containing 10% FBS and 10^4^ HUVECs were poured in each well of96-well plates (SPL) and kept for 24 days. Next, the exhausted medium was replaced with 200 fresh culture medium supplemented with 2% FBS. After completion of the experimental period, 50 μl MTT (dilution: 5 mg/ml, 3- (4, 5-dimethylthiazol-2-yl) -2, 5-diphenyltetrazolium bromide; Cat no: M5655.100MG; Sigma-Aldrich) was added to each well and kept in an incubator for the next 4 h. After the formation of formazan crystal, 100-μl diethyl sulfoxide (Merck; Darmstadt; Germany) solution was added to dissolve the crystals and generate a blue-to-purple appearance. Cell viability was measured by reading optical density at 570 nm wavelength by using BioTek microplate reader and expressed as % of the control group. This assay was performed octuplicate [16].

### Measuring the levels of inflammatory cytokines

In this research, the content of paracrine inflammatory factors such as NF-ƙB, TNF-α, and IL-6 was measured. For this purpose, supernatants from different groups were collected and subjected to the ELISA assay (IL-6; Cat no: E0090Hu, TNF-α; Cat no: E0082Hu, NFƙ-B; Cat no: E0690Hu). Supernatants were centrifuged at 400 g for 5 min to exclude the cell debris. After that, the contents of the cytokines were measured according to the manufacturer’s instruction. This assay was performed in triplicate [17].

### Measuring pro-inflammatory status by using immunofluorescence staining

We also performed immunofluorescence staining (IF) to detect the intracellular level of TNF-α after exposure to the DHA, Pal, and insulin. In brief, 1 × 10^4^ cells were placed in each well of 8 well slide chambers (SPL). After reaching 70–80% confluence, cells were subjected to the experimental protocol at the respective time point. Cells were fixed with 4% PFA and permeabilized using permeabilizing buffer (Ref no: 00–0055-56; eBioscience; USA) for 20 min. Cells were then incubated with anti-TNF-α antibody (dilution: 1:100; Cat no: ab34674; Abcam) for 1 h followed by PBS wash (3 × 10 min). After that, we added FITC-conjugated secondary antibody (1: 1000; eBioscience; USA) for 1 h at RT. After PBS wash, the nuclei were stained with 1 μg/ml for 30 s [18].

### Evaluation of NO content by Griess assay

The intracellular level of NO was measured using the Griess assay, as previously described (Ref). HUVECs (5 × 10^4^cells/well) were plated in each well of 96-well plates and enrolled in the experimental procedure as those mentioned above. After completion of treatment, 200 μl supernatant was mixed with 15 μl of Griess A solution and maintained at 37̊C for 20 min. Then, we added 15 μl of Griess B reagent to samples, and the optical density (OD) was determined at 450 nm using a microplate reader. NO content was expressed in nM. The final NO contents were calculated after comparison with standard control (Sodium nitrite) [19].

### Flow cytometry analysis of apoptosis

To measure the occurrence of apoptosis in cells from different groups, the percent of HUVECs entering apoptosis was determined by using flow cytometry analysis. For this purpose, 2× 10^5^ HUVECs were seeded in each well of 24-well plates and allowed to reach 70–80% confluence. The cells were subjected to the treatment protocol. After 48 h, cells were detached using 0.25% Trypsin-EDTA solution and washed twice with PBS. After that, cells were permeabilized using a permeabilizing buffer (eBioscience, USA) at room temperature for 30 min. In the current experiment, we incubated cells from different groups with 2.5 μl FITC-tagged Annexin-V antibody at 4̊C for 30 min. After twice washing with PBS, we added 2.5 μl propidium iodide and incubated cells for b5 minutes. The cells were analyzed by using the BD FACSCalibur® system and FlowJo software ver.7.6.1 [20].

### PCR array

To investigate the effect of DHA on atherosclerotic changes in HUVECs under treatment with hyper insulin concentration, we performed PCR array analysis. After completion of the experimental procedure, RNAs were extracted from each group by using an RNA extraction kit (Cat no.11828665001, Roche) followed by cDNA synthesis (Cat no. YT4500, Yekta Tajhiz Azma). Using the RT2 Profiler PCR Array, we monitored the expression of 86 genes participating in the atherosclerosis signaling pathway. Raw data were processed using the 2^−ΔΔCT^ method to determine statistical significance between the groups [21].

### Statistical analysis

Data are presented as mean ± SD. One-way ANOVA and Tukey postdoc analysis was used to find statistically significant differences between the groups. *P* < 0.05 was considered statistically significant. All experiments were repeated three times unless mentioned.

## Results

### DHA decreased intracellular pal accumulation revealed by oil red O staining

**Fig. 1:**
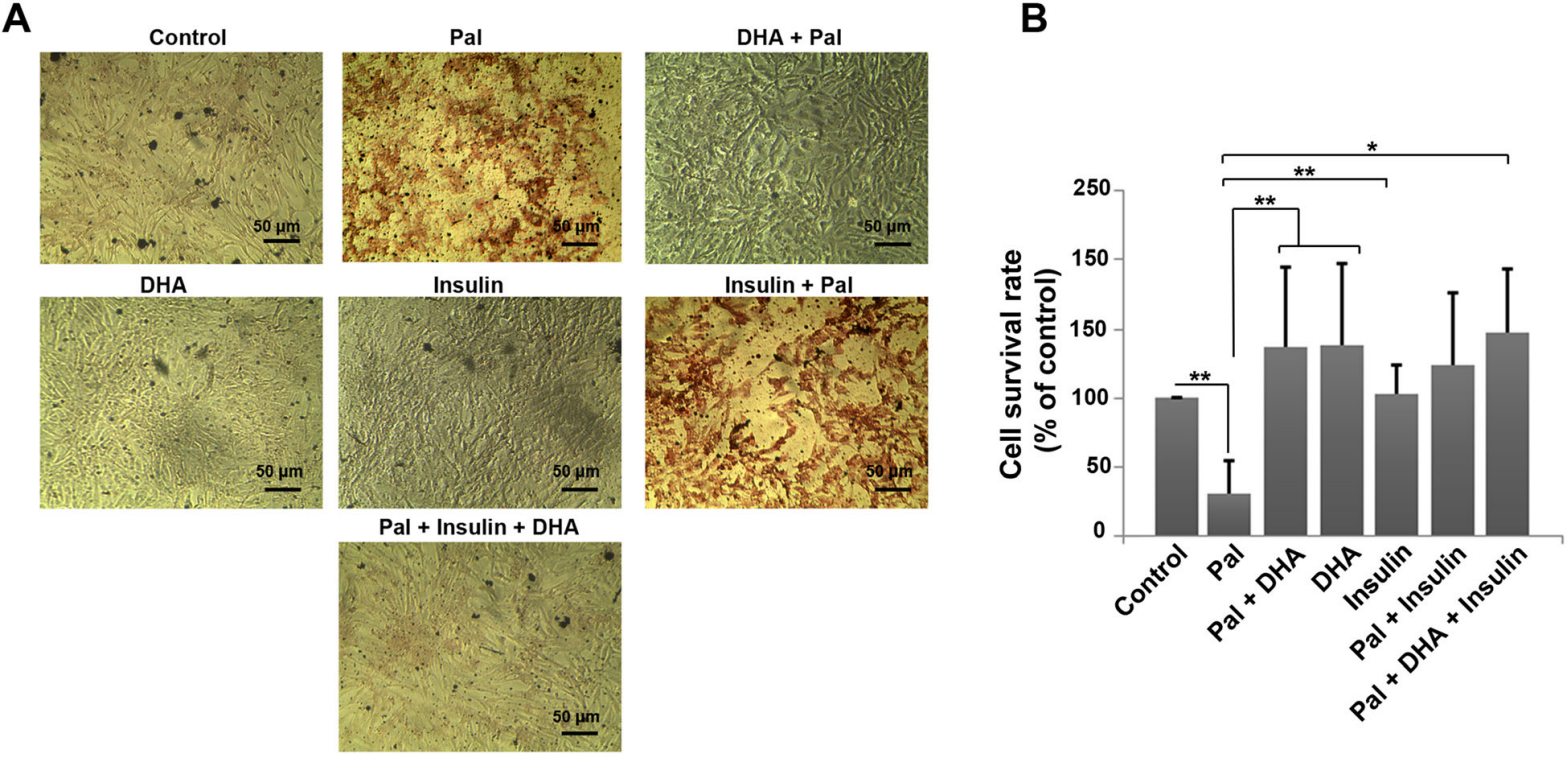
Measuring Effect of DHA on intracellular accumulation of Pal inside HUVECs evaluated by Oil Red O staining (**a**). The addition of DHA to the culture medium could decrease the intensity of Pal inside HUVECs. The presence of Pal acid appears a red-colored appearance. MTT assay (**b**). MTT assay showed that Pal decreased HUVECs viability compared to the control group. The addition of DHA and insulin increased cell survival rate and closed to near-to-control levels (*n* = 3). One-Way ANOVA and Tukey posthoc analysis. *p < 0.05; and **p < 0.01

To confirm atherosclerotic changes in HUVECs after being-incubated with 1 mM Pal, we performed Oil red O staining (Fig. 1a). Data from Oil red O staining results showed a large number of red-colored cells in the Pal group, indicating the efficiency of our protocol to induce atherosclerotic changes in HUVECs after 48 h (Fig. 1a). According to bright-field imaging, we noted that DHA could decrease the accumulation of Pal in the HUVECs 48 h after the addition of DHA. These data showed that DHA could revert Pal-induced atherosclerotic changes in HUVECs. In this experiment, we did not find the red-colored cells in insulin and DHA groups. The co-treatment of HUVECs with 50 μM insulin and 1 mM Pal contributed to the promotion of atherosclerotic features. The addition of high-dose insulin did not inhabit the initiation of atherosclerotic changes. Interestingly, incubation of Pal + Insulin HVECs with 50 μM DHA could reduce the accumulation of Pal inside the cells and closed it to near-to-control levels (Fig. 1a).

### Effects of insulin and DHA on the viability of atherosclerotic HUVECs

We performed an MTT assay to show the possible therapeutic effect of DHA on ECs after exposure to 50 μM insulin and 1 mM Pal. MTT showed that Pal decreased the HUVECs survival rate compared to the control group (*p* < 0.01; Fig. 1b). Based on our data, the treatment of HUVECs with 50 μM insulin did not yield statistically significant differences in viability rate compared to the control cells (*p* > 0.05). In cells received50 μM DHA, HUVECs viability was increased after being-exposed to Pal over 48 h (*p* < 0.01; Fig. 1b). Similarly, we found that the combination of insulin with Pal decreased the detrimental effects of Pal and closed it to near-to-normal levels (*p*_*Control* vs. *Pal + Insulin*_ *>* 0.05). In the group received DHA + Pal plus insulin, we found a maximum survival rate compared to the other groups. The addition of Insulin and DHA increased HUVECs viability compared to the Pal group (*p* < 0.01; Fig. 1b).

### DHA decreased inflammation of HUVECs after treatment with pal

**Fig. 2:**
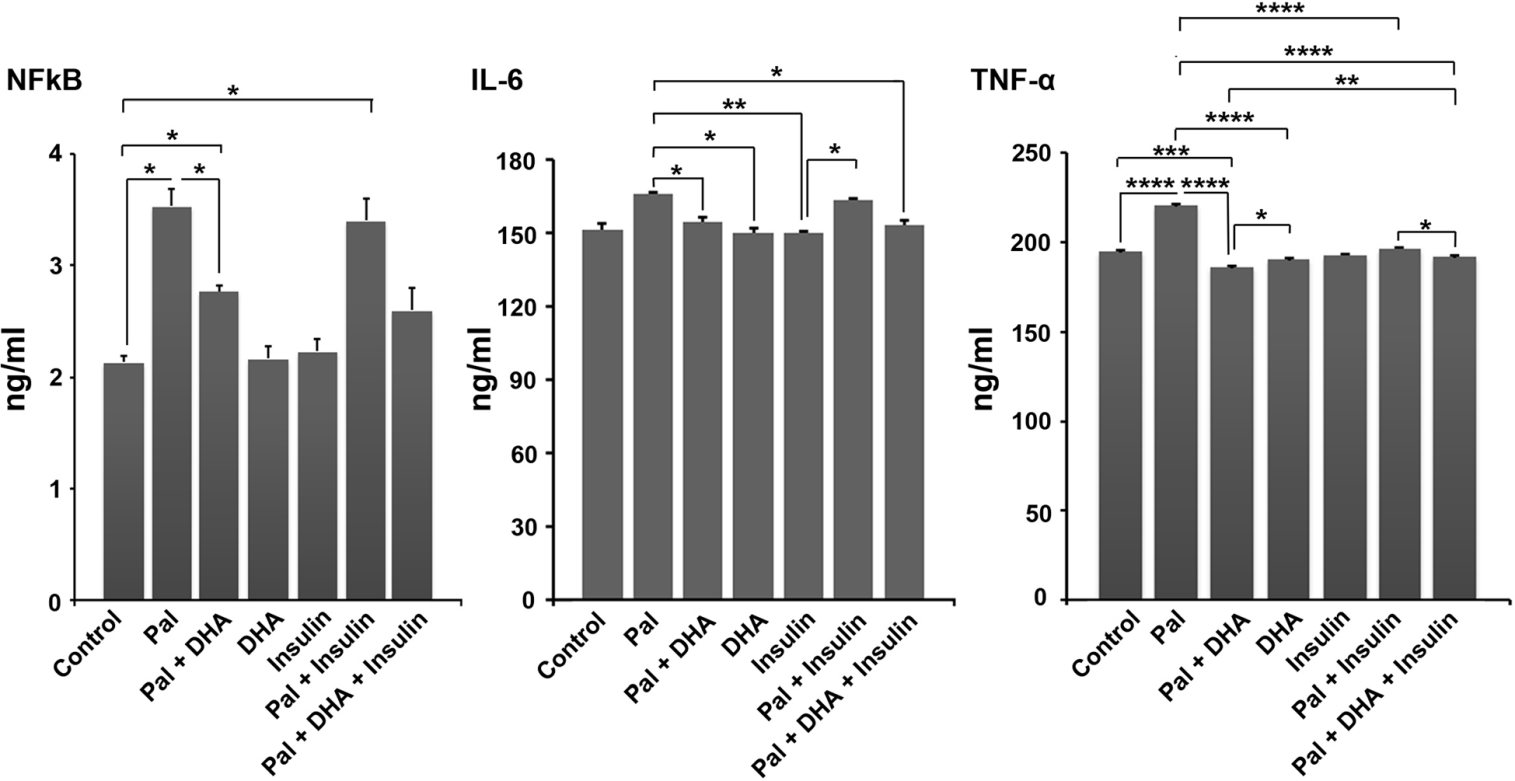
The effect of insulin and DHA on the pro-inflammatory cytokines produced by HUVECs after being exposed to the Pal. One-Way ANOVA and Tukey posthoc analysis (*n* = 3). *p < 0.05; **p < 0.01; ***p < 0.001; and *p* < 0.0001

**Fig. 3:**
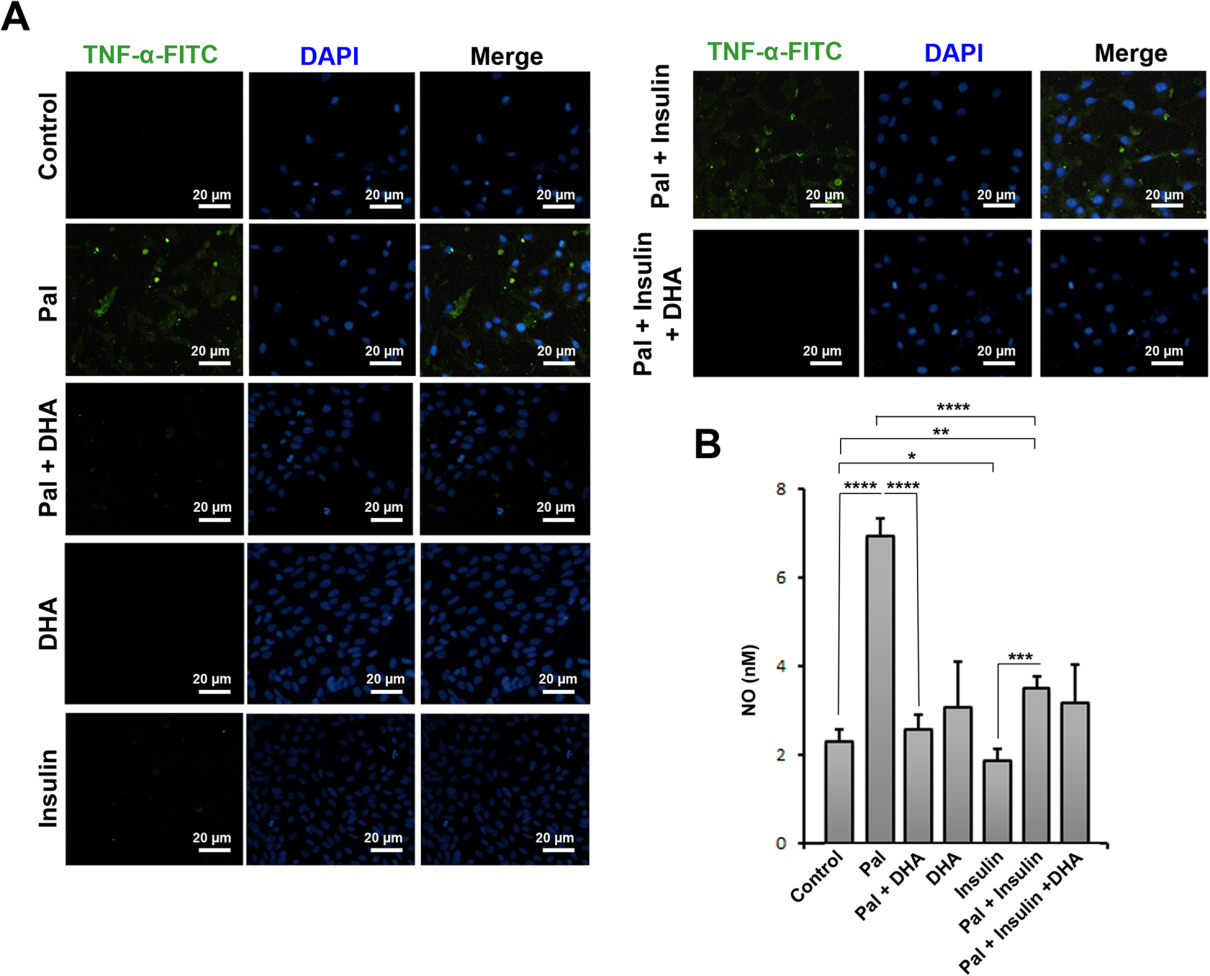
Measuring TNF-α protein levels inside the HUVECs using immunofluorescence staining (**a**). Data revealed that Pal induced the synthesis of TNF-α in HUVECs 48 h, indicated by green-colored appearance. The addition of Insulin and DHA decreased the TNF-α levels showing a reduced inflammatory response. Detection of NO and nitrosative status using Griess assay (**b**; *n* = 3). According to our data, Pal induced nitrosative stress in HUVECs by the increase of NO. The addition of DHA and insulin reduced NO production in these cells and returned to near-to-control levels. One-Way ANOVA and Tukey posthoc analysis (*n* = 3). *p < 0.05; **p < 0.01; ***p < 0.001; and *p* < 0.0001

To monitor the anti-inflammatory Effect of DHA on Pal-treated HUVECs, we measured the levels of NF-ƙB, TNF-α, and IL-6 after 48 h (Fig. 2). Data showed that incubation of HUVECs with 1 mM Pal induced the synthesis of pro-inflammatory cytokines compared to the non-treated control cells (*p* < 0.05; Fig. 2). Treatment of the Pal group with 50 μM DHA decreased the inflammatory status by diminishing protein levels of all cytokines compared to the Pal group (*p* < 0.05; Fig. 2). Compared to the control, co-incubation of HUVECs with Insulin and Pal increased significantly the protein levels of NF-ƙB (*p* < 0.05; Fig. 2), while we found statistically non-significant differences in the levels of IL-6 and TNF-α in Pal + Insulin compared to the control cells. Treatment of the Pal + Insulin group with 50 μM DHA inhibits inflammatory response in HUVECs compared to the Pal group. IF staining confirmed that 48-h incubation of HUVECs with Pal increased intracellular levels of TNF-α, which appeared as green-colored cells (Fig. 3a). The addition of Insulin to Pal-treated cells decreased TNF-α levels.

### DHA decreased nitrosative stress in HUVECs after treatment with pal

Monitoring the levels of NO could reflect nitrosative stress in ECs [22]. In this study, the levels of NO were measured using the Griess assay. Based on our data, incubation of HUVECs with 1 mM Pal increased significantly NO levels compared to the control cells (*p* < 0.0001; Fig. 3b). According to our data, DHA had the potential to decrease nitrosative stress in HUVECs by diminishing NO levels compared to the Pal + DHA group (*p* < 0.0001; Fig. 3b). Co-treatment of HUVECs with Pal and Insulin decreased significantly NO compared to the Pal group (*p* < 0.01; Fig. 3b). These data showed that insulin and DHA could decrease nitrosative stress in HUVECs exposed to 1 mM Pal, showing therapeutic effects of Insulin and DHA on atherosclerotic cells.

### DHA *and Insulin decreased apoptotic changes in atherosclerotic HUVECs*

**Fig. 4:**
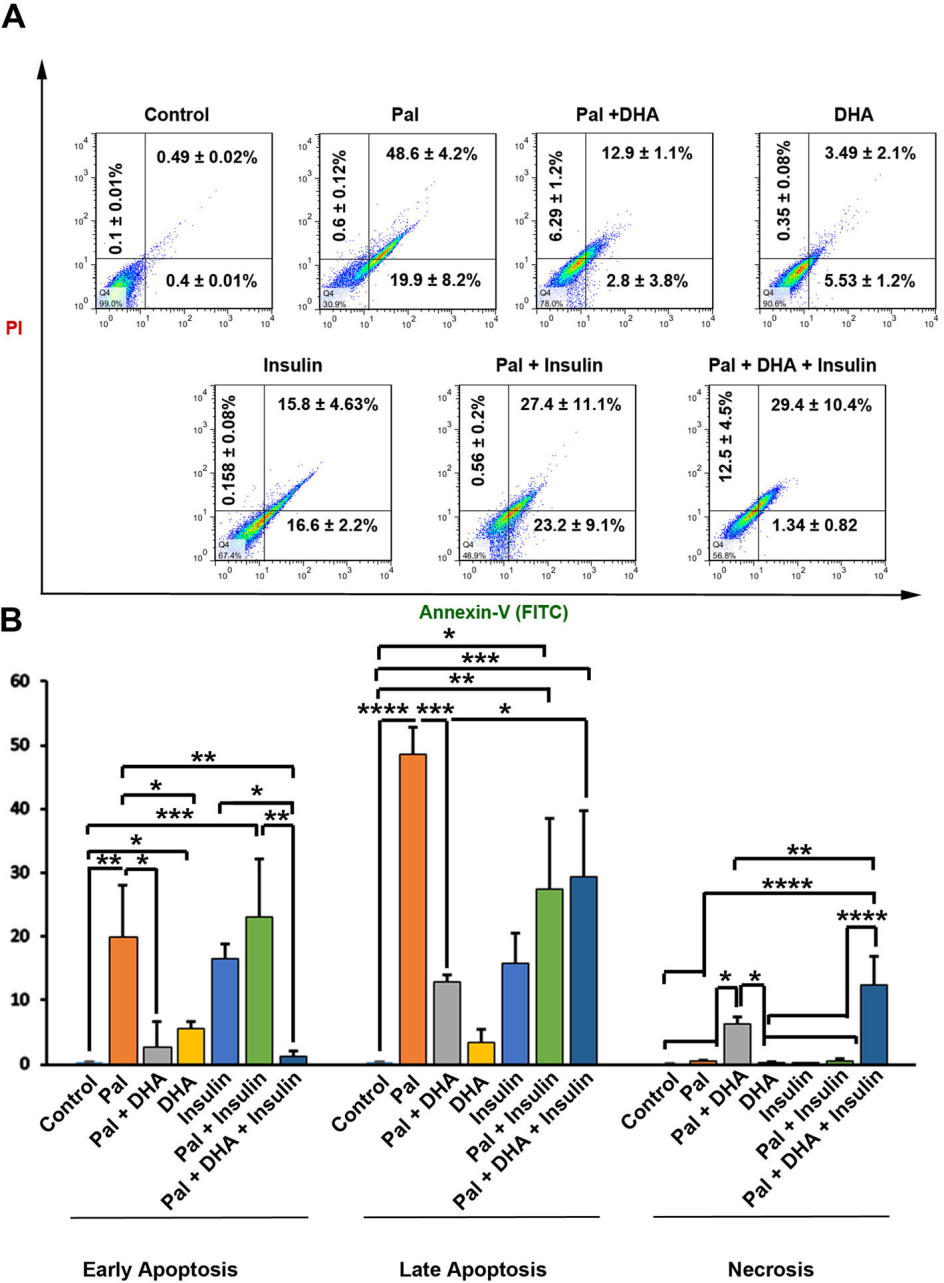
Calculating the percent of apoptotic and necrotic HUVECs using flow cytometry analysis of HUVECs (**a** and **b**). According to our data, the percent of early and late apoptotic cells were reduced in Pal-treated group after treatment with DHA and Insulin. One-Way ANOVA and Tukey posthoc analysis (*n* = 3). *p < 0.05; **p < 0.01; ***p < 0.001; and *p* < 0.0001

Flow cytometry analysis showed that incubation of HUVECs with 1 mM Pal increased the expression of Annexin-V compared to the control cells, showing induction of both early and late apoptosis (Fig. 4a-b). The addition of DHA to the Pal group decreased the percent of apoptotic cells significantly compared to the Pal group. Besides, Insulin could not alter the number of early and later apoptotic cells compared to Pal-treated HUVECs (*p* > 0.05; Fig. 4a-b). Data showed that the addition of DHA and Insulin to the Pal group could inhibit the number of early apoptotic cells. At the same time, these values were increased in terms of necrotic and later apoptosis. It seems that the addition of DHA and insulin could inhibit the apoptosis in early stages rather than later progressive cell injury. We also found that Pal + DHA and Pal + DHA + Insulin increased the number of necrotic cells compared to the other groups.

### DHA and insulin changed the expression of genes related to atherosclerosis signaling pathway

PCR array analysis was performed to elucidate the expression of different genes related to various signaling atherosclerosis transduction in HUVECs after being exposed to the DHA, Insulin, and Pal (Table 1). Data analysis showed that the exposure of HUVECs to 1 mM Pal could change the expression of genes participating in coagulation. In this regard, we noted the up-regulation of FGA (2.3-fold) and down-regulation of LPA (0.19) in Pal-treated cells compared to the control group. Compared to the control group, the expression of genes correlated with circulation singling transduction such as APOB (0.24-fold), ELN (0.14), and LPA (0.19) was down-regulated after 48-h incubation with 1 mM Pal. According to our data, Pal was not able to modulate the expression of PDGFA, PDGFB, PDGFRB, and vWF in terms of platelet activation as compared with control cells (*p* < 0.05; Table 1). We also found the addition of 1 mM Pal induced the expression of ACE (2.6-fold) and FGA (2.3-fold). We also monitored the transcription of different genes related to EC capacity to the dynamic composition of the extracellular matrix. The expression of LPA (0.19-fold), ELN (0.14-fold), PLIN2 (0.43-fold), APOB (0.24-fold), CCL-2 (0.42-fold), IL-1A (0.27-fold), and TNC (0.07-fold) was decreased in group received 1 mM Pal while the expression of ACE (2.6-fold). MMP-3 (6.5-fold) and FGA (2.3-fold) increased compared to the control HUVECs. We also found that Pal can alter the expression of genes involved in lipid transport and metabolism (*p* < 0.05; Table 1). Data showed that the expression of genes such as LDLR, FABP3, PPARA, PTGS1, APOB, FABP3, LPA, NR1H3, and PLIN2 was significantly decreased in condition enriched with the 1 mM Pal. In contrast, the expression of ABCA was increased in atherosclerotic conditions compared to the control cells. Treatment of HUVECs with μDHA alone or in combination with Pal reduced expression of FGA compared to the control group (*p* < 0.05). It was notified that insulin was to alter the expression of FGA in the presence of 1 mM Pal. DHA did not change the expression of FGA in cells pre-treated with Insulin and Pal. The addition of DHA alone or, in combination with Pal, reduced the LPA compared to the control. It seems that the treatment of HUVECs with DHA increased the expression level of ELN, but no-significant differences were found between the Pal, DHA, and DHA + Pal groups. No statistically significant differences were found in the group exposed to Insulin alone or in combination with Pal. We noted that DHA could decrease elevated levels of MMP-3 induced by Pal compared to the control levels (*p* < 0.05). DHA alone reduced the expression of ACE while co-treatment of HUVECs with DHA and Pal contributed to prominent activation of ACE (126.1-fold), showing the synergistic Effect of DHA and Pal on the expression of ACE in HUVECs after 48 h. We found non-significant differences in the expression of PLIN2 in all groups treated with DHA compared to the pal group. The addition of DHA and Insulin to Pal-treated HUVECs increased the expression of APOB compared to the control cells. Insulin was unable to alter the expression of CCL-2 while the treatment of HUVECs with DHA increased CCL-2 expression in all groups and close the transcription levels to near-to-control levels. The expression of CSF-2 was increased in all groups received 50 μM DHA compared to the control group. Similar to Pal-treated HUVECs, Insulin alone suppressed the expression of CSF-2. Insulin decreased the expression of ABCA1 while the addition of Pal and DHA increased the activity of these genes compared to the control group. According to our data, the expression of LDLR was increased in all groups received Pal, DHA, and the combination of LDLR. Insulin alone or in combination with DHA and Pal increased the levels of LDLR and closed it to the near-to-control levels. Pal decreased the level of NR1H3, the addition of Insulin, and DHA increased expression rate, resulting in statistically non-significant differences compared to the control HUVECs.

**Table 1:** PCR array analysis of genes participating in atherosclerosis in HUVECs after being exposed to DHA, Insulin and Pal

Gene	Pal		Pal + DHA		DHA		Ins		Ins + Pal		Ins + Pal+ DHA	
	Fold change	***p*** Value	Fold change	***p*** Value	Fold change	***p*** Value	Fold change	***p*** Value	Fold change	***p*** Value	Fold change	***p*** Value
FGA	2.2623	0.000195	0.4162	0.000423	0.226	0.000122	0.9886	0.858718	2.7311	0.00034	1.2056	0.07855
ITGA2	0.5148	0.186216	0.0097	0.041603	0.013	0.041974	0.7513	0.393342	0.0295	0.043892	0.0209	0.04288
LPA	0.1957	0.00154	0.036	0.000765	0.0639	0.000857	1.1497	0.286114	0.7457	0.081279	0.0684	0.00088
SERPINE1	0.2362	0.128603	0.0244	0.07897	0.018	0.077837	0.6983	0.389053	0.034	0.080701	0.5395	0.2671
APOA1	0.47	0.39454	1.4	0.6013	1.07	0.51948	0.37	0.37705	2.44	0.91345	1.28	0.57166
APOB	0.24	0.00579	2.04	0.00380	0.64	0.06326	2.31	0.35248	6.02	0.00648	6. 86	0.25711
APOE	0.73	0.42647	2.03	0.60903	2.2	0.63731	0.3	0.37543	0.56	0.41406	6.06	0.65791
COL3A1	0.1461	0.111593	0.5637	0.291867	0.3664	0.1846	0.6983	0.396531	0.8568	0.797511	0.6857	0.38775
ELN	0.1418	0.004155	0.8408	0.310959	0.3837	0.013332	0.9063	0.507802	1.4722	0.059493	0.3476	0.01136
ENG	0.2109	0.050585	0.1698	0.044254	0.2252	0.053022	0.7955	0.421651	0.773	0.3946	5.8257	0.00036
LPL	0.0889	0.209023	0.6485	0.423387	0.7043	0.453014	0.5671	0.383148	0.3377	0.287539	0.4796	0.34411
NPY	0.3719	0.100227	0.4571	0.130198	0.5653	0.183136	1.5873	0.323059	2.1488	0.064041	1.1366	0.95138
PDGFA	0.3081	0.30454	0.2267	0.279675	0.2845	0.297106	0.527	0.381798	0.9322	0.569464	0.3387	0.31475
PDGFB	0.4482	0.15356	0.0418	0.057159	0.0368	0.056512	1.7634	0.332943	1.4482	0.695445	0.0681	0.06075
PDGFRB	0.1927	0.189131	0.1563	0.177984	0.1907	0.188486	0.6286	0.389363	0.5817	0.362109	0.4479	0.29033
VWF	0.1548	0.349314	0.2082	0.356684	0.2419	0.361405	0.3421	0.375786	0.9787	0.479183	0.2582	0.36382
ACE	2.6606	0.001328	126.1722	0.000001	1.3167	0.181824	1.2456	0.288333	2.8606	0.019744	3.5848	0.0008
SERPINB2	0.1631	0.273307	0.0315	0.240553	0.0392	0.242367	0.5069	0.379783	0.0439	0.243517	0.1127	0.26037
MMP1	1.2506	0.485206	0.0451	0.013016	0.0273	0.01227	1.3371	0.323544	0.0452	0.013028	0.0483	0.01316
MMP3	6.5663	0.000196	0.0976	0.044903	0.203	0.060289	1.5801	0.32596	0.2478	0.068863	0.1284	0.04895
FN1	0.2159	0.131015	0.0238	0.103147	0.0311	0.104081	2.9369	0.346906	0.1113	0.11499	0.2895	0.1439
PLIN2	0.4311	0.000402	0.5082	0.000732	0.5443	0.00106	1.0809	0.272634	0.6299	0.003951	0.3108	0.0002
CCL2	0.4282	0.005346	2.6829	0.000356	1.2678	0.0937	0.9466	0.615357	1.0125	0.949646	2.4055	0.00157
CCL5	0.2591	0.374313	0.2057	0.369794	0.1308	0.363532	0.2654	0.374848	1.8657	0.60549	0.6885	0.41259
CSF2	0.2711	0.025064	8.9048	0.000026	1.4678	0.133968	0.854	0.451294	0.3974	0.064759	3.0769	0.00183
CTGF	0.2734	0.185923	5.6427	0.00221	1.0231	0.734643	0.661	0.392441-	0.7044	0.427377	2.5402	0.10558
FGF2	0.0655	0.329726	0.231	0.354905	0.1689	0.345261	0.3622	0.376065	0.1476	0.34208	0.8251	0.45984
HBEGF	0.0439	0.350439	0.1364	0.359529	0.1107	0.35698	0.2901	0.375072	1.2918	0.49116	0.2825	0.37437
IFNAR2	0.1335	0.108671	0.034	0.0873	0.0395	0.088357	0.6919	0.390488	0.0684	0.094137	0.4674	0.2339
IFNG	0.4542	0.113708	0.0841	0.035513	0.014	0.029131	1.5025	0.337465	0.8758	0.489667	1.2179	0.81462
IL1A	0.2709	0.016371	0.0605	0.007017	0.039	0.006489	0.8658	0.439692	1.0208	0.966917	0.591	0.08553
IL2	0.5464	0.172261	0.0619	0.041536	0.0276	0.03794	1.5729	0.33996	0.3901	0.106421	0.9203	0.54866
IL3	0.0619	0.063493	0.054	0.064519	0.0808	0.066625	0.7327	0.396901	0.3571	0.139598	0.1053	0.071
IL4	0.2308	0.070085	0.0373	0.039986	0.03	0.038965	0.7634	0.398858	0.0346	0.039484	0.041	0.0402
IL5	0.0346	0.003306	0.0138	0.003061	0.0342	0.003302	0.8838	0.434694	0.0347	0.003309	0.0687	0.00377
LAMA1	0.1693	0.11724	0.0677	0.100878	0.0136	0.093215	2.5196	0.346648	0.0217	0.09433	0.0184	0.09388
LIF	0.2517	0.3286	0.2234	0.321968	0.1412	0.303453	0.4522	0.379005	0.2603	0.330848	0.1065	0.29603
SPP1	0.2781	0.126554	0.03	0.08018	0.0098	0.077336	2.1127	0.345463	0.0358	0.081029	0.0235	0.07926
THBS4	0.0522	0.319942	0.0953	0.32688	0.0409	0.318128	0.3809	0.376427	0.1226	0.331415	0.0294	0.31632
TNC	0.0708	0.005068	0.0141	0.004107	0.0028	0.003942	0.8738	0.42883	0.5234	0.043917	0.0119	0.00408
VEGFA	0.1601	0.115629	0.0772	0.102267	0.0239	0.094585	2.5196	0.34609	0.0267	0.094995	0.0319	0.09572
ABCA1	14.0178	0.000009	20.159	0.205066	3.49	0.000098	1.841	0.390056	0.4623	0.003394	7.7525	8.8E-05
LDLR	0.365	0.038722	0.2613	0.025061	0.2554	0.024471	1.331	0.30633	0.8292	0.389805	0.3487	0.03658
FABP3	0.1018	0.008043	0.6334	0.103746	0.8515	0.390203	1.2744	0.300601	3.9597	0.000488	1.7359	0.03217
PPARA	0.1982	0.007923	0.2181	0.008623	0.1571	0.006657	1.2399	0.297656	2.6329	0.001847	0.1282	0.00595
PTGS1	0.0477	0.022809	0.2276	0.041649	0.5146	0.124771	0.8139	0.429567	0.3359	0.062454	0.3187	0.05851
MSR1	0.1335	0.274678	0.4269	0.357643	0.4969	0.380583	0.4966	0.380455	0.2963	0.318531	0.5042	0.38355
NR1H3	0.2146	0.083775	0.482	0.182761	0.4682	0.175494	0.7548	0.411917	0.8595	0.557452	1.9049	0.12631
PPARD	0.081	0.318945	0.0657	0.316328	0.0708	0.317195	0.3951	0.376967	0.2024	0.340457	0.2002	0.34006
PPARG	0.7855	0.320083	0.0445	0.09	0.0142	0.085664	2.2941	0.341156	0.1837	0.113502	0.0115	0.08528
RXRA	0.0533	0.331931	0.1001	0.338489	0.2671	0.362909	0.3524	0.375971	0.15	0.345694	0.4269	0.38787

The *p* values are calculated based on a Student’s t-test of the replicate 2^(−Delta Ct)^ values for each gene in the control group and treatment groups, and p values less than 0.05 are indicated in red for genes with 2 fold increase and blue for genes with −2 fold decrease (*n* = 3)

## Discussion

Atherosclerosis is one of the most important causes of cardiovascular disease is caused by local inflammation in the luminal surface of blood vessels after prolonged metabolic diseases such as diabetes mellitus [23]. In individuals with diabetes mellitus, the levels of insulin increase soon after the onset of the hyperglycemic condition [24]. To the best of our knowledge, there are few reports regarding the inhibitory/stimulatory effect of hyperinsulinemia in diabetic patients with atherosclerosis. The current experiments aimed to explore the effect of insulin in high doses on atherosclerotic HUVECs in vitro. Also, the beneficial therapeutic effects of DHA were explored.

Our results showed that the current protocol is eligible to induce atherosclerotic conditions in human ECs indicated by red-colored appearance via Oil Red O staining [25]. The incubation of HUVECs with Pal + Insulin also increased the intracellular accumulation of Pal after 48 h. However, the levels of Pal were less in Pal + Insulin compared to the Pal group. One reason would be that the incubation of cells with Insulin increases the influx of Pal to the inside of the ECs while simultaneously accelerates lipid biogenesis and metabolism [26]. Consistent with our data, previous experiments demonstrated that Insulin has the potential to activate the function of cell-membrane bond lipoprotein lipase and increase the entrance of Pal [27]. The lack of intracellular Pal accumulation in HUVECs exposed to DHA may be related to an increased lipolysis capacity and activation of peroxisome proliferator-activated receptor-gamma [28]. These data showed an inhibitory effect of DHA to prohibit the accumulation of fatty acid in endothelial lineage via the promotion of lipolysis and lipid metabolism. We also noted that the incubation of HUVECs with Pal decreased cell viability compared to the normal cells [17, 28]. It has been demonstrated that the exposure of endothelial EA.hy926 cells with Pal decreased cell survival via the reduction of mitochondrial activity leading to accumulation of intracellular and mitochondrial superoxide contents [29]. In this study, we found that the addition of DHA and insulin increased cell viability in Pal-treated cells. Maillard and co-workers showed that the induction of mitochondrial activity and phosphorylation of MAPK in DHA-treated cells coincided by increased proliferation and steroidogenesis rate in bovine granulosa cells [30]. It seems that the DHA reduces fatty acids toxicity in ECs via the mitochondrial consumption and conversion into final bioproducts under atherosclerotic condition in ECs. According to our data, both early and late apoptotic changes were found in the Pal group. In support of our data, Ulloth and co-workers showed the activation of Caspase signaling in PC12 cells exposed to Pal [31]. DHA decreased the apoptotic changes in Pal-treated cells while insulin had neutral effects of prohibiting the promotion of early and later apoptosis.

Along with these data, we also measured the pro-inflammatory status by monitoring the levels of NF-ƙB, TNF-α, and IL-6. Here, the decrease of NF-ƙB, TNF-α, and IL-6 was shown in the DHA + Pal group compared to the Pal cells. According to our data, insulin did not alter the levels of IL-6 and NF-ƙB, but not TNF-α, in Pal-treated cells. Commensurate with these descriptions, there are several mechanisms related to the therapeutic effects of DHA on apoptosis and multiple physiological roles [30]. It has been shown that DHA and other n-3 polyunsaturated fatty acids could affect the production of different cytokines that coincided with simultaneous synthesis inflammation resolving metabolites such as maresins, resolvins, and protectins [32, 33]. DHA increases the bioactivity of sensory molecules such as peroxisome proliferator-activated receptors and NF-ƙB by which control the biosynthesis of other cytokines. On the other hand, DHA could stimulate cell division and viability by the activation of certain signaling pathways and effectors, mainly MAPK1/3 and Akt [30]. Both DHA and insulin decreased the nitrosative stress in HUVECs pretreated with Pal. Previous experiments confirmed the potency of DHA to regulate oxidative stress [34]. Also, it has been shown that DHA has the potential to increase the activity of Heme Oxygenase 1 and Glutathione peroxidase in macrophages, leading to reduced oxidative stress [22]. In the current experiment, we showed that Insulin alone or, in combination with Pal, decreased NO levels in Pal-treated HUVECs. Previously, it has been shown that Insulin could suppress the activity of inducible nitric oxide synthase, PGE2, and cyclooxygenase in alveolar macrophages [35]. We also performed PCR array analysis to monitor the expression of different genes participating in the atherosclerosis signaling pathway. According to our data, DHA and Insulin could alter genes from different singling transduction pathways. Data showed that the levels of MMP-1 and -3 were increased in atherosclerotic HUVECs. It seems that both DHA and insulin could blunt increased levels of MMP-1 and -3 in Pal-treated cells. It has been shown that the formation of atherosclerotic plaques was initiated as a result of cellular migration followed by alternation in situ levels of MMP-1 and MMP-3 [36, 37]. Also, the incubation of HUVECs with Pal and DHA increased the production of ECM components by the up-regulation of genes such as CCL-2 and CTGF and down-regulation of specific genes such as IL-4, showing the regression of atherosclerotic condition [38, 39]. The increase of ABCA1 in Pal plus DHA treated HUVECs showed the activation of lipid efflux mechanism to increase cell resistance to high levels of Pal [40]. These changes were coincided with the increase APOB in HUVECs after being incubated with Insulin and DHA. These data showed that DHA and Insulin did not improve dysregulation of lipid metabolism in HUVECs. Taken together, it seems that DHA and Insulin could alter the expression of both negative and positive atherosclerotic genes and further experiments are highly demanded to show the underlying mechanisms.

## Conclusion

Here, we showed that DHA is eligible to decrease the atherosclerotic changes in human endothelial lineage induced by Pal. The addition of Insulin could improve the regenerative potential of DHA in the atherosclerotic condition. Further investigations are needed to address the underlying mechanisms.

## Data Availability

The datasets used and analyzed during the current study are available from the corresponding author on reasonable request.
